# Impact of macrophages on Balamuthia mandrillaris virulence properties using human brain microvascular endothelial cells in vitro

**DOI:** 10.1186/1742-4690-9-S1-P38

**Published:** 2012-05-25

**Authors:** Abdul Matin, Khalid Mehmood, Suk-Yul Jung

**Affiliations:** 1 Institute of Biomedical and Genetic Engineering, Islamabad, Pakistan

## Introduction

*Balamuthia* amoebic encephalitis (BAE) is a serious human disease almost always leading to death. An important step in BAE is amoebae invasion of the bloodstream, followed by their haematogenous spread. *Balamuthia mandrillaris* entry into the central nervous system (CNS) most likely occurs at the blood–brain barrier (BBB) sites. Macrophages are thought to be the first line of defense in many infectious diseases and are present in high numbers during infections. The objective of the present study was to determine the impact of cytokines and macrophages on the virulence characteristics of *B. mandrillaris in vitro*.

## Materials and methods

*In vitro*, *B. mandrillaris* were used to demonstrate the effects of cytokines and macrophages on the physiological and morphological characteristics of amoeba. Using human brain microvascular endothelial cells (HBMEC), which constitutes the BBB, adhesion and cytotoxicity assays were performed. To investigate the engulfing property and proteolytic activity of the amoeba, phagocytosis and zymography assays were conducted respectively.

## Results

It was observed *B. mandrillaris* exhibited >90 % binding and >70 % cytotoxicity to HBMEC which was further enhanced in the presence of cytokines and macrophages. It has also been observed that cytokines TNF-α and TGF-β significantly increased the *B. mandrillaris* numbers in the presence of macrophages. It is important to note that amoebic numbers were more than doubled in the presence of cytokines and macrophages within 24h. We have shown in the past the bacteria uptake by *B. mandrillaris* is limited which is further significantly inhibited in the presence of cytokines during phagocytosis assays. Zymography assays revealed that cytokines and macrophages have no inhibitory effect on proteolytic activity of *B. mandrillaris*. In addition the activated macrophages did not show any vital effects on amoebic virulence properties.

## Conclusion

Overall we described for the first time that cytokines and macrophages has no inhibitory effects on the virulence properties of *B. mandrillaris in vitro*.

